# Evaluating anemia on non-contrast thoracic computed tomography

**DOI:** 10.1038/s41598-022-24265-8

**Published:** 2022-12-10

**Authors:** Bita Abbasi, Maliheh Seyed Hosseini, AmirAli Moodi Ghalibaf, Reza Akhavan, Maryam Emadzadeh, Ehsan Bolvardi

**Affiliations:** 1 Department of Radiology, Faculty of Medicine, Mashhad University of Medical Sciences, Mashhad, Iran; 2 Student Research Committee, Faculty of Medicine, Birjand University of Medical Sciences, Birjand, Iran; 3 Department of Emergency Medicine, Faculty of Medicine, Mashhad University of Medical Sciences, Mashhad, Iran; 4 Clinical Research Development Unit, Ghaem Hospital, Mashhad University of Medical Sciences, Mashhad, Iran

**Keywords:** Haematological diseases, Diagnosis

## Abstract

Anemia is a major global disease burden factor linked to an adverse impact on overall prognosis and negatively affects the quality of life. There are some suggested findings for anemia on non-contrast chest CT, like relatively dense interventricular septum (septal sign) or fairly dense aortic wall (aortic ring sign). The measured attenuation value is a reproducible physical density measurement, readily obtainable from a standard CT examination. There is no reliable cut-off for blood attenuation to suggest anemia on the non-contrast chest CT. In the current study, we evaluated subjective and objective criteria’ diagnostic accuracy for diagnosing anemia on unenhanced thoracic CT. This study is approved by Mashhad University of Medical Sciences. The patients admitted in the internal medicine ward of our hospital from June 2019 to March 2020 for whom a non-contrast chest CT was acquired for any non-traumatic medical indication, were enrolled in this retrospective study. For the subjective assessment, the radiologists were asked to record the presence or absence of the “aortic ring sign” and “interventricular septum sign”. For the objective evaluations, blood density was measured at various anatomic locations. A total of 325 patients were included in this study. There was a significant correlation between blood attenuation in all measured segments and Hb level (0.78 (R^2^: 0.61), p = 0.000). Findings revealed that considering the aortic arch threshold value as 20 HU is the best diagnostic performance for detecting severe anemia. Subjective analysis revealed that the aortic ring sign was more sensitive (82.5%) than the interventricular septum sign (32%) in detecting anemia, whereas the latter character was more specific (87% and 99.2%, respectively). The results suggest that it is possible to detect anemia from an unenhanced chest CT scan. Both objective and subjective criteria show promising sensitivity and specificity.

## Introduction

Incidental findings on thoracic computed tomography (CT) scans are common; some of them have a considerable impact on patients’ management and outcome^1,2^. Incidental detection of anemia on unenhanced thoracic CT has been previously reported. Anemia is defined as a reduced absolute number of circulating red blood cells, represented in clinical practice by a low serum hemoglobin (Hb) concentration^3^; in detail, anemia is defined as Hb concentration less than 12 g/dL and 14 g/dL for females and males, respectively^4^. It is a major global disease burden factor linked to an adverse impact on overall prognosis and quality of life^5–7^. In routine practice, anemia is diagnosed by a laboratory test of serum Hb using a peripheral blood sample^8^. There might not be enough time to wait for peripheral blood test analysis in acute conditions, especially in trauma patients. Making the diagnosis on the CT scan, which is commonly requested in these settings, might be of great help. There are some suggested findings for anemia on non-contrast chest CT, like relatively dense interventricular septum (septal sign), differences between the interventricular septum and left ventricle cavity (IVS-LV) in CT attenuation, or relatively hyperattenuating aortic wall (aortic ring sign)^9–12^. These findings are subjective and reader-dependent and subject to significant inter-observer variability.

Furthermore, the findings may lead to a false impression of anemia in some conditions like glycogen or iron storage diseases^13,14^. The objective analysis provides a more accurate evaluation of anemia. The measured attenuation value is a reproducible physical density measurement, readily obtainable from a standard CT examination. There is no reliable cut-off for blood attenuation to suggest anemia on the non-contrast chest CT. The current study evaluated the diagnostic accuracy of subjective and objective criteria for diagnosing anemia on an unenhanced thoracic CT scan.

## Materials and methods

This retrospective study was carried out according to relevant guidelines and regulations. The Ethics committee of Mashhad University of Medical Sciences (MUMS) approved this retrospective observational study. The review board at MUMS waived informed consent due to the observational nature of the research, under the waiver statement MUMS.MEDICAL.REC.1398.405.

### Patient selection

Consecutive patients admitted to the internal medicine department of our academic hospital in the period between June 2019 and March 2021 were candidates to be enrolled in this study. The patients were chosen from those for whom a non-contrast chest CT was acquired for any non-traumatic medical indication including dyspnea, suspected interstitial lung disease (ILD), or those who were undergoing metastasis workup. Other inclusion criteria included complete blood count (CBC) evaluation within 24-h of unenhanced thoracic CT scan. Exclusion criteria included the known history of iron overload or glycogen storage disease, repetitive blood product transfusion or active bleeding at the time of CT scan, recent intravenous contrast administration, and significant artifact in the chest CT scan. Clinical assessment, laboratory investigations, and unenhanced thoracic CT scans were reviewed for each patient.

Anemia was defined as hemoglobin (Hb) level below 12 g/dL for women and below 14 g/dL for men. Anemia was further categorized as mild (Hb below the normal level, and more than 9.5 g/dL), moderate (8 < Hb < 9.4 g/dL) and severe (Hb < 8 g/dL)^15,16^.

### CT scan analysis

All images were acquired using Neusoft™ 16-slice CT scanner with the following parameters: 180–450 mAs, 120 kV, and section thickness of 1 mm. All images were reconstructed using a soft tissue standard kernel filter specified by the manufacturer. Selected patients did not receive oral contrast before or during the CT examination.

The images were evaluated by a radiologist with 10 years of experience, who was unaware of laboratory findings. The images were interpreted using soft tissue window (window width, 200 HU, window level, 75 HU) on a medical monitor. For the subjective assessment, the radiologist was asked to record the presence or absence of “aortic ring sign”, defined as the presence of hyperattenuating aortic wall against relatively hypodense blood pool, and “interventricular septum sign” defined as hyperattenuating interventricular septum against relatively hypodense blood pool. For the objective assessments, blood density was measured by placing two circular regions of interest (ROI) approximately 1 cm^2^ over each of the following structures: ascending aorta at the level of main pulmonary artery bifurcation, descending aorta, aortic arch, main pulmonary artery at the level of bifurcation, left ventricular cavity, right ventricular cavity and inferior vena cava (IVC). The interventricular septum was not included in the measurements, as this structure is not readily visible on most non-contrast chest CT scans. We had asked the readers to declare a positive interventricular septal sign only whenever they were both utterly positive on the presence of this sign and exclude the cases of unequivocal findings. The ROI size was adjusted based on the anatomic structure being evaluated. The ROIs were selected so that areas of beam hardening or motion artifact and vessel walls were avoided. The mean HU in each region was recorded. All ROI measurements were performed using a dedicated workstation (Neusoft™).

### Statistical analysis

Using the IBM SPSS Statistics for Windows, Version 25.0. Armonk, NY: IBM Corp, we analyzed our data. The relations of HU numbers with Hb level were evaluated by linear regression analysis. In addition, in selected cases with a high R-value group correlation coefficient, receiver operating characteristic (ROC) analysis for the diagnosis of anemia was performed. The subjective and objective analyses' sensitivity, specificity, and accuracy were calculated. p-value < 0.05 was considered statistically significant.

## Results

A total of 325 patients were included in this study. The mean age was 64.2 ± 17.1 years (ranged from 18 to 98 years) without significant gender difference (mean age for men was 63 years and for women was 66 years) (p: 0.27). The mean Hb level was 11.8 g/dL (ranging from 4 to 19.8) in our study group 194 patients (59.7%) were anemic. Hb value for the anemic group ranged from 4 to 13.8 g/dL (mean 9.6 ± 2.3 g/dL). Corresponding value for the non-anemic group ranged from 12 to 19.8 g/dL (mean 14.9 ± 1.7 g/dL). Forty-five patients (13.8%) had severe anemia, whereas moderate and mild anemic were reported in 42 (12.9%) and 107 (32.9%) patients, respectively. (Demographic and laboratory findings are summarized in Table 1.

**Table 1:** Demographic and laboratory findings in 325 studied patients.

	Total	Non-anemic subjects	Anemic subjects	p value
Age: mean ± SD (range)	64.2 ± 17.1 (18–98)	59.7 (17.9)	67.7 (15.6)	0.048*
Sex (female frequency))	34.2%	36.6%	32.6%	0.27**
Hb level (g/dL): (IQR)	9.3–14.4	14–15.8	8–11.4	0.000***
Hct level (g/dL): (IQR)	27.8–40.3	40–45.3	25–35	0.000***

*Hb* Hemoglobin, *Hct* hematocrit, *SD* standard deviation, *IQR* interquartile range.

*t-student test, **Chi-square test, ***Mann–Whitney test.

The mean CT density (Hounsfield value) was measured in the aortic arch, ascending aorta, descending aorta, pulmonary artery, inferior vena cava, right and left ventricular cavities (Supplementary Information).

The data derived from this analysis revealed a significant difference between the blood density in various anatomic locations in anemic and nonanemic patients (Table 2).

**Table 2:** Mean blood attenuation in various anatomic locations.

	Total	Nonanemic subjects	Anemic subjects	p value*
**Hounsfield unit (interquartile range)**				
Aortic arch	28–43	40–47.5	25–36	0.000
Ascending aorta	27–44	39.7–50	24–37	0.000
Descending aorta	29–45	39.2–50	25–37	0.000
Pulmonary artery	30–42	40–47.7	27–37	0.000
IVC	30–45	40–50	28–37	0.000
Right ventricle	34–45	35–47	27.2–38	0.000
Left ventricle	32–45	35–48	27–37.7	0.001

*IVC* inferior vena cava.

*Mann Whitney test.

The correlation between the measured densities and serum Hb level was evaluated. Correlation coefficients of various sites of HU measurement and serum Hb level are summarized in Table 3.

**Table 3:** Correlation coefficients of various CT attenuation values with serum Hb level.

	Aortic arch	AA	DA	PA	IVC	RV	LV
Total	0.78	0.73	0.74	0.73	0.73	0.58	0.50
Male	0.82	0.75	0.77	0.75	0.77	0.61	0.52
Female	0.74	0.74	0.72	0.66	0.62	0.41	0.40
Age > 60 years	0.79	0.76	0.78	0.75	0.74	0.63	0.55
Age ≤ 60	0.72	0.58	0.55	0.62	0.63	0.49	0.41

*CT* computed tomography, *Hb* hemoglobin, *AA* ascending aorta, *DA* descending aorta, *IVC* inferior vena cav.

**Figure 1:**
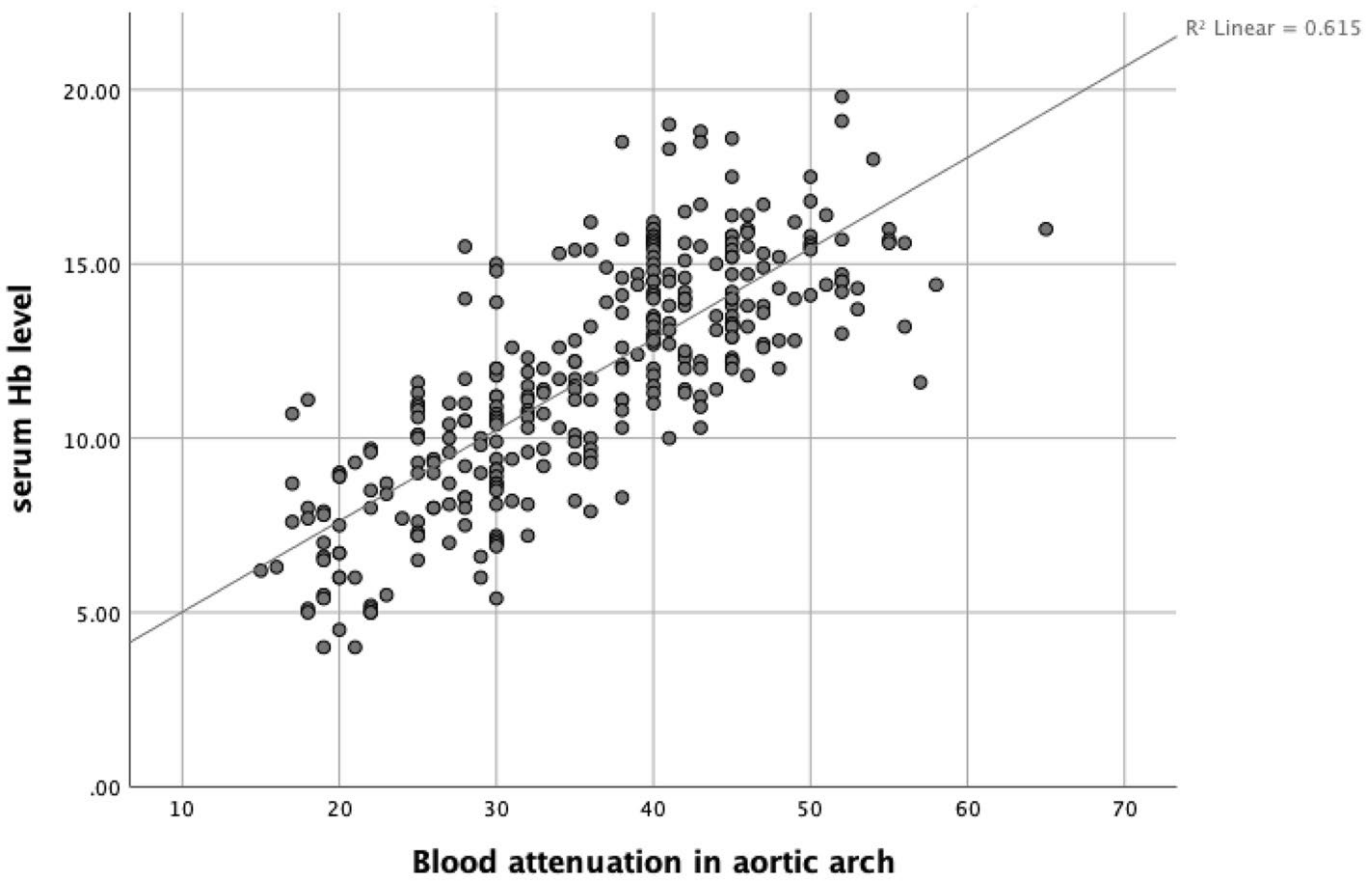
Relation between the blood attenuation in the aortic arch and serum Hb level in 325 subjects. The correlation coefficient was 0.78 (R^2^: 0.61). *Hb* hemoglobin.

The most significant correlation coefficient was seen between the blood attenuation in the aortic arch and serum Hb level (Fig. 1). The level of correlation between the blood attenuation and ascending and descending aorta, pulmonary artery, and IVC were also statistically significant. The correlation between the blood attenuation and Hb level was less significant in the right and left ventricles.

Among the 325 subjects, the correlation between blood attenuation and Hb level in the aortic arch, pulmonary artery, and IVC was more significant in male subjects than females. The mentioned correlations were also more significant in patients older than 60.

**Figure 2:**
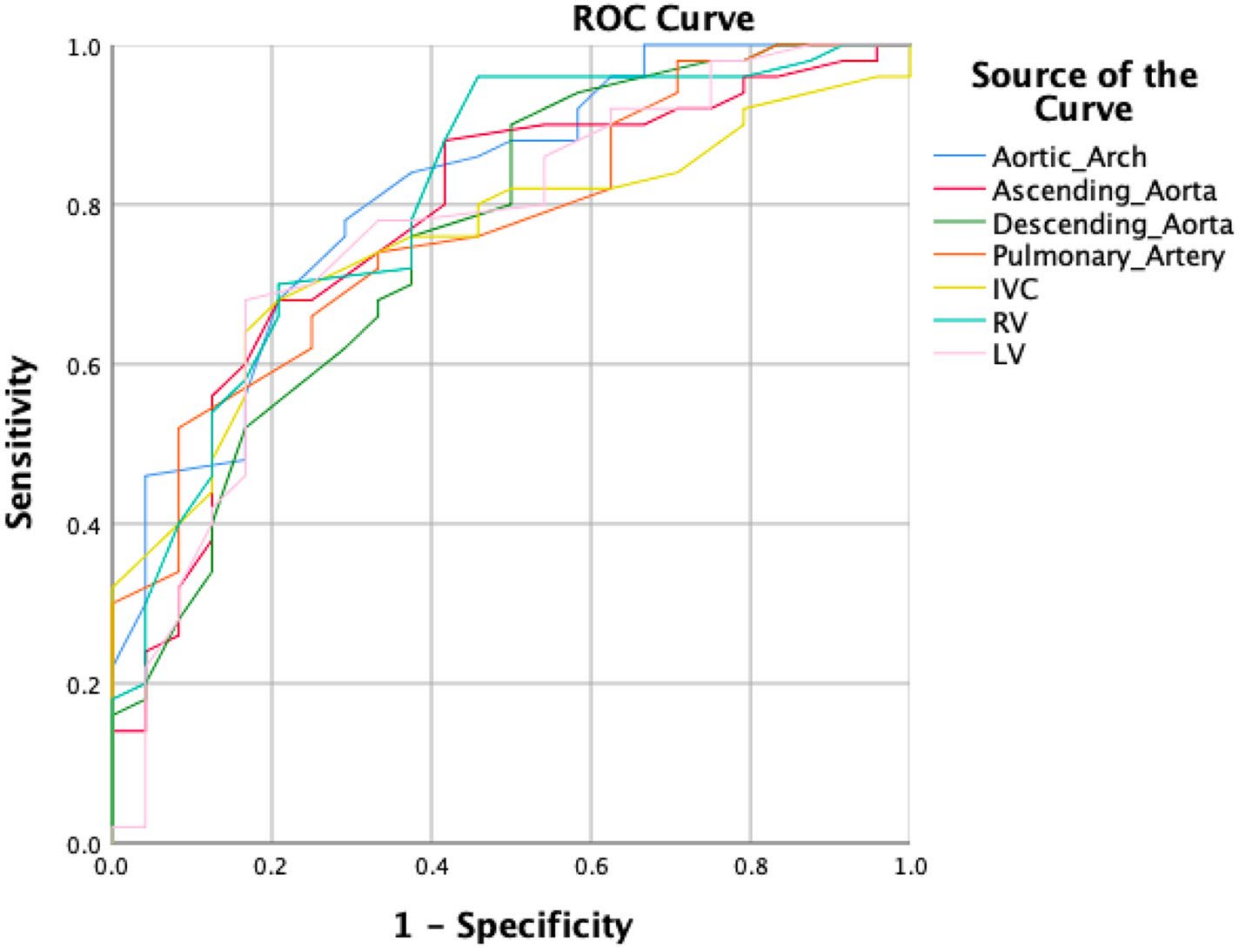
Results obtained using receiver operating characteristic (ROC) analysis. The area under the curve was 0.816 for the aortic arch, the highest among the other anatomic locations of blood attenuation measurement. *IVC* inferior vena cava, *RV* right ventricle, *LV* left ventricle.

The results of the ROC analysis are shown in Fig. 2. The area under the ROC curve was 0.816 for the HU value in the aortic arch, 0.775 in the ascending aorta, 0.759 in the descending aorta, 771 in the pulmonary artery, 0.755 in the IVC, 0.806 in the right ventricular cavity, and 0.765 in the left ventricular cavity.

Table 4 shows the sensitivity, specificity, and accuracy for the diagnosis of anemia based on various threshold values of blood attenuation.

**Table 4:** Sensitivity, specificity, and accuracy for diagnosis of anemia at various threshold values of blood CT attenuation in different anatomic locations.

Threshold	location	Sensitivity	Specificity	Accuracy
26	Aortic arc	33	100	60
	Ascending aorta	36	98.5	61.2
	Descending aorta	32.5	100	52.7
	Pulmonary artery	21.6	99.2	52.9
	IVC	21.6	97	52
	LV	3	99	41.8
	RV	2.6	97.7	49
35	Aortic arc	73	91.6	86
	Ascending aorta	72.7	85.5	78.8
	Descending aorta	70.6	85.5	76.6
	Pulmonary artery	68.6	87	76
	IVC	72.2	87	78.1
	LV	7.7	89.3	40.6
	RV	7.7	89.3	40.6
45	Aortic arc	96.4	34.3	71.4
	Ascending aorta	94.3	39.7	72.3
	Descending aorta	94.9	42	73.5
	Pulmonary artery	96.9	34.3	71.7
	IVC	95.9	48.9	76.9
	LV	11.3	72	35
	RV	11.8	69.5	35.1

*RV* right ventricle, *LV* left ventricle.

The best diagnostic performance in the objective CT attenuation evaluation was for the aortic arch blood attenuation using the threshold of 35 as the cut-off value.

**Figure 3:**
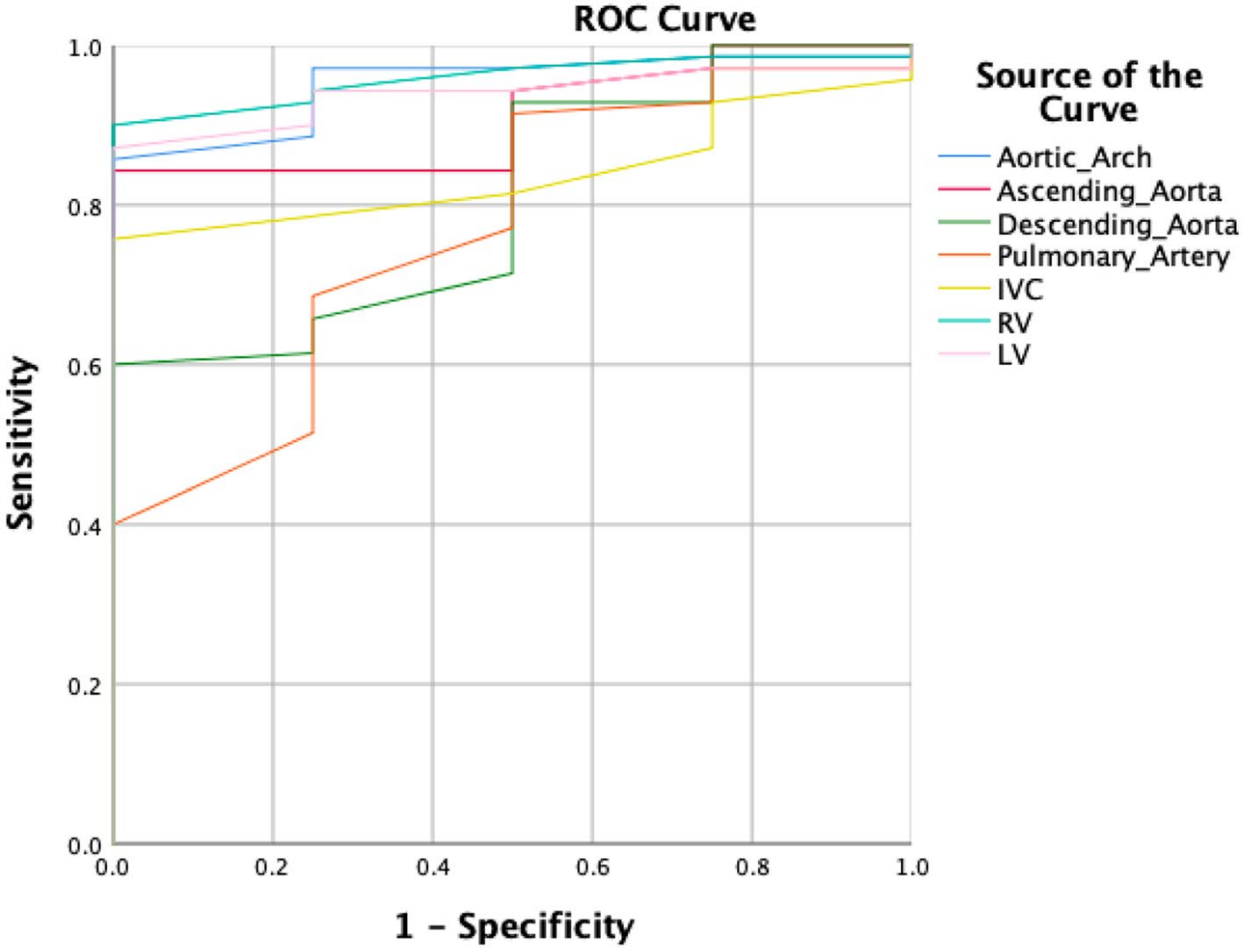
Results obtained using receiver operating characteristic (ROC) analysis to diagnose severe anemia. *IVC* inferior vena cava, *RV* right ventricle, *LV* left ventricle.

Figure 3 shows the ROC analysis for the performance of blood attenuation measurement in various anatomic parts to diagnose severe anemia.

The area under the ROC curve was 0.952 for the HU value in the aortic arch, 0.911 in the ascending aorta, 0.805 in the descending aorta, 0.77 in the pulmonary artery, 0.839 in the IVC, 0.959 in the right ventricular cavity, and 0.939 in the left ventricular cavity.

Table 3 shows the sensitivity, specificity, and accuracy for diagnosing severe anemia based on various threshold values of blood attenuation. The best diagnostic performance for detecting severe anemia was for the aortic arch when using 20 HU as the threshold value.

Aortic ring sign was reported in 122 (37.5%, 105 anemic and 117 non-anemic patients) and ventricular septum sign was reported in 26 (8%, all in anemic) patients. The sensitivity, specificity and accuracy of these signs for anemia, moderate/severe anemia and severe anemia are summarized in Table 5. Subjective analysis revealed that the aortic ring sign was more sensitive than the interventricular septum sign in detecting anemia, whereas the latter was more specific (Table 5). The frequency of false-positive readings for aortic ring sign was 38.2% in patients ≤ 50 years old and 61.8% in patients over 50 years. This difference was statistically significant (p: 0.000, chi-square test). The interventricular septum sign was falsely positive in just one patient without anemia. The aortic ring sign was falsely negative in 2 cases of moderate and just one case of severe anemia (Table 6). Table 7 summarizes the best diagnostic performances of subjective and objective criteria.

**Table 5:** Sensitivity, specificity, and accuracy for diagnosis of severe anemia at various threshold values of blood CT attenuation in different anatomic locations.

Threshold (HU)	Aortic arch			Ascending aorta			Descending aorta			Pulmonary artery			IVC		
	Sens. (%)	Spec. (%)	Accu. (%)	Sens. (%)	Spec. (%)	Accu. (%)	Sens. (%)	Spec. (%)	Accu. (%)	Sens. (%)	Spec. (%)	Accu. (%)	Sens. (%)	Spec. (%)	Accu. (%)
20	44.4	97.1	89.9	35.6	97.1	88.6	26.7	98.6	88.6	8.9	97.5	85.2	31.1	97.5	88.3
26	73.3	88.9	86.7	71.1	85.7	83.7	73.3	89.3	87.1	53.3	93.2	87.7	46.7	91.1	85
30	97.8	61.1	66.1	100	58.9	64.6	97.8	60	65.2	93.3	61.4	65.8	100	60	65.5

**Table 6:** Diagnostic accuracy of aortic ring sign and interventricular septum sign for different severities of anemia.

	Anemia			Moderate/severe anemia			Severe anemia		
	Sens. (%)	Spec. (%)	Accu. (%)	Sens. (%)	Spec. (%)	Accu. (%)	Sens. (%)	Spec. (%)	Accu. (%)
Aortic ring sign	82.5	87	84	96.5	61	70.5	97.8	52.5	58.8
Interventricular septum sign	32	99.2	59	69	98.7	90.8	77.8	90	88.3

**Table 7:** Performance of three diagnostic parameters (aortic ring sign, interventricular septum sign, and aortic arch CT attenuation value) in diagnosing anemia.

Diagnostic parameters	Sensitivity (%)	Specificity (%)	Accuracy (%)
Aortic ring sign	82.5	87	84
Interventricular septum sign	32	99.2	59
Aortic arch CT attenuation value ≤ 35 for anemia	73	91.6	86
Aortic arch CT attenuation value ≤ 20 for severe anemia	44.4	97.1	89.9

## Discussion

**Figure 4:**
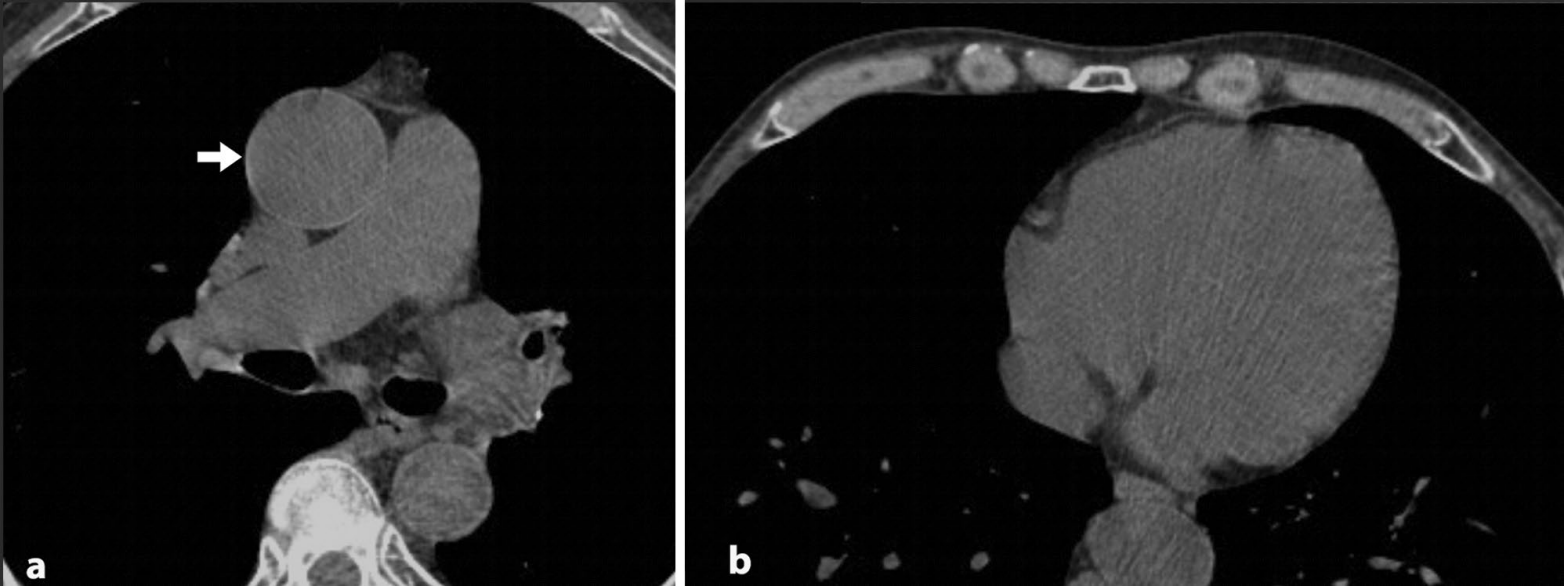
In this 74 y/o man with mild anemia, aortic ring sign is present (arrow in (**a**)), but there is no interventricular septal sign (**b**).

**Figure 5:**
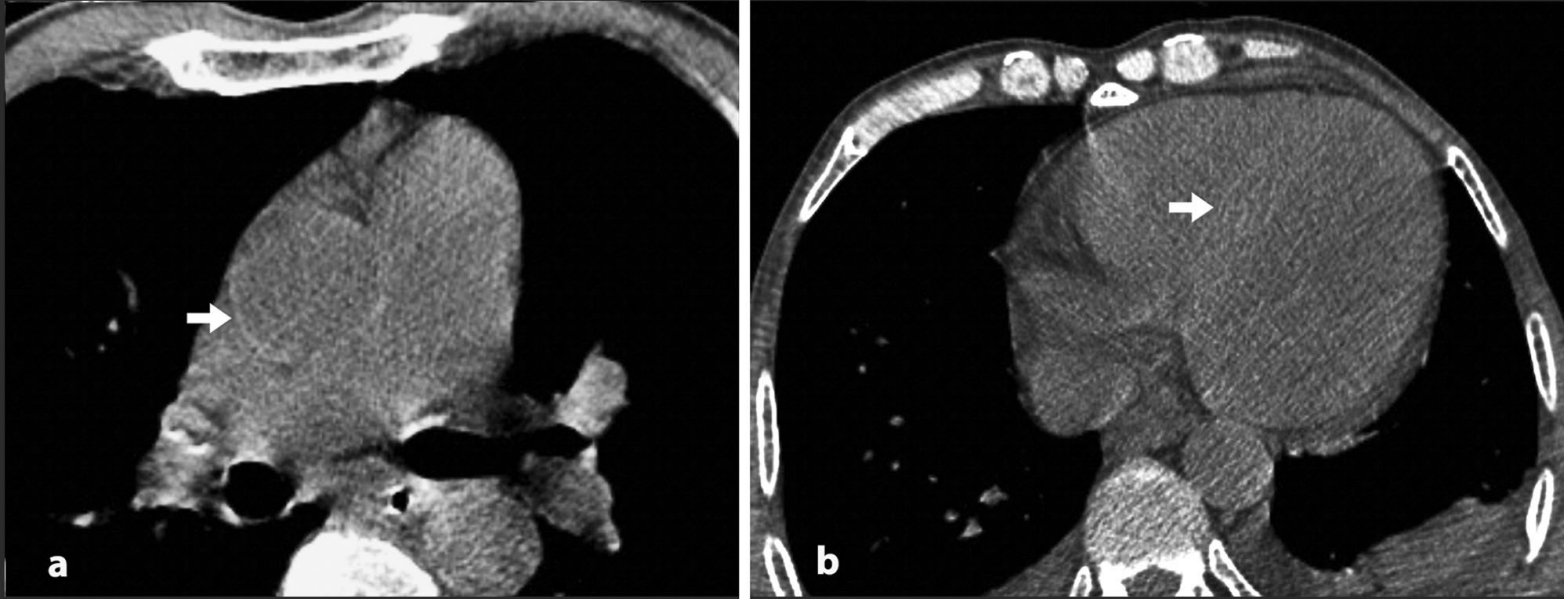
In this 67 y/o man with severe anemia, both aortic ring sign (arrow in (**a**)) and interventricular septum sign (arrow in (**b**)) were present.

**Figure 6:**
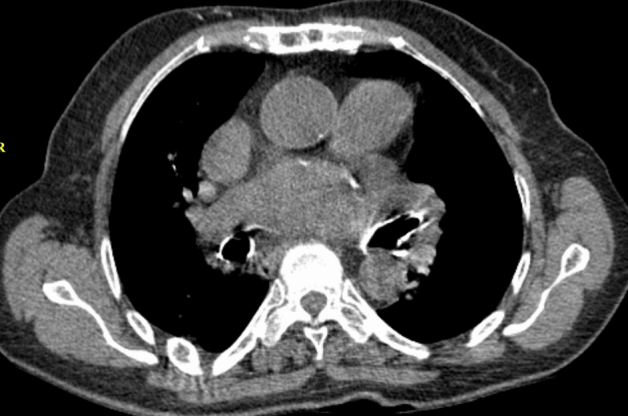
The presence of aortic ring sign in a nonanemic patient. This finding is attributed to the fine mural calcifications in the aortic wall.

Anemia remains a neglected and underdiagnosed entity that carries an increased risk of mortality, morbidity, and hospital stay^17^. It is essential to recognize every means helpful in the early detection of anemia. There was a significant correlation between blood attenuation and hemoglobin level in the current study on non-contrast chest CT scans of 325 subjects. Besides the objective measurements, subjective evaluations showed acceptable sensitivity and specificity in detecting anemia. The interventricular septum sign had an excellent specificity of 99.1% in detecting anemia. Still, the sensitivity of this sign was very low (32%) in detecting anemia and was not acceptable for moderate and severe anemia (69% and 77.8%, respectively). We had asked the readers to declare a positive interventricular septal sign only whenever they were both utterly positive on the presence of this sign and exclude the cases of unequivocal findings. This, and the subjectivity of the sign could have led to the increased frequency of false-negative readings (Fig. 4). There was only one case of false-positive IVS, meaning this sign could reliably diagnose anemia on a non-contrast chest CT scan (Fig. 5). False-positive interventricular septum sign has been suggested to be secondary to iron on glycogen deposits in the myocardium. Hence, there was no suggestive medical history in our patient with a false-positive interventricular septum sign. Aortic ring sign had a modest sensitivity (82.5%) for diagnosing anemia and optimal sensitivity for diagnosing moderate and severe cases of anemia (96.5% and 97.8%, respectively). The better sensitivity of the aortic ring sign could be due to greater contrast between the aortic wall, which is mainly composed of collagen and elastin fibers and blood pool. There is less contrast between myocardial and blood pool attenuation, making the interventricular septum sign less sensitive. The lower specificity of the aortic ring sign could be due to the presence of false-positive readings in the setting of early calcium depositions in the aortic wall that make the aortic wall denser on CT scan (Fig. 6). This study’s statistically higher frequency of false-positive aortic ring sign readings in the elderly population supports this hypothesis. While not applicable in diagnosing mild cases of anemia, aortic ring sign performed well in ruling out moderate and severe cases with sensitivities of 96.5% and 97.8%, respectively. The aortic ring sign was falsely negative in two circumstances of moderate and just one case of severe anemia.

Data derived from the objective analysis revealed a significant correlation between blood pool attenuation in various anatomic locations and serum hemoglobin levels. There was a significant difference between blood CT density in different anatomic areas between anemic and nonanemic subjects. The aortic arch showed the best diagnostic performance, while the lowest accuracy was seen in the left and right ventricular cavities. The decreased accuracy for the ventricular cavities could be due to the combination of heart pulsations and respiratory movements, leading to motion artifact and less accurate densitometries, while the aortic arch is located transversely within the chest making this structure less a subject to the artifacts mentioned above. The best diagnostic accuracy was returned with the blood attenuation cutoff of 35 in the aortic arch with the sensitivity, specificity, and accuracy of 73%, 91.6%, and 86%, respectively. Our results concur with those previously derived from the literature and show that blood CT attenuation can be a valuable adjunct for diagnosing anemia. Considering the subjective analysis, the presence of interventricular septum sign can reliably diagnose anemia, and the absence of aortic ring sign is a reliable sign for ruling out anemia. Thus, a rapid inspection for these two findings can be implemented in the routine chest CT scan evaluation.

This study has some limitations. We asked just one radiologist to subjectively evaluate the images regarding the presence of aortic ring sign and interventricular septal sign. The validity would have increased if we had asked more than one radiologist to interpret the images, and reported the kappa of agreement between them. Another limitation of this study is that we did not include the patient’s body mass indices (BMI) in our evaluation. Although tube current and voltage settings were the same in all CT examinations, different BMIs could have resulted in different image qualities. Quantum mottling artifact in obese patients may have affected the analysis, especially the subjective evaluation.

## Conclusion

It is possible to detect anemia from an unenhanced chest CT scan. Both objective and subjective criteria show promising sensitivity and specificity.

## Supplementary Information

Supplementary Information.

## Data Availability

The datasets used and/or analyzed during the current study is available from the corresponding author on reasonable request.
